# Gut-Microbiome Implications in Opioid Use Disorder and Related Behaviors

**DOI:** 10.3389/adar.2022.10311

**Published:** 2022-03-15

**Authors:** Bridget Herlihy, Sabita Roy

**Affiliations:** ^1^ Department of Surgery, University of Miami Miller School of Medicine, Miami, FL, United States; ^2^ Department of Neuroscience, University of Miami Miller School of Medicine, Miami, FL, United States

**Keywords:** opioid use disorder, substance use disorder, microbiome, behavior, gut-brain, withdrawal

## Abstract

Substance use disorder (SUD) is a prevalent disease that has caused hundreds of thousands of deaths and affected the lives of even more. Despite its global impact, there is still no known cure for SUD, or the psychological symptoms associated with drug use. Many of the behavioral consequences of drug use prevent people from breaking the cycle of addiction or cause them to relapse back into the cycle due to the physical and psychological consequences of withdrawal. Current research is aimed at understanding the cause of these drug related behaviors and therapeutically targeting them as a mechanism to break the addiction cycle. Research on opioids suggests that the changes in the microbiome during drug use modulated drug related behaviors and preventing these microbial changes could attenuate behavioral symptoms. This review aims to highlight the relationship between the changes in the microbiome and behavior during opioid treatment, as well as highlight the additional research needed to understand the mechanism in which the microbiome modulates behavior to determine the best therapeutic course of action.

## The Microbiome and Behavior

There is a well-studied relationship between the gut microbiome and mental health disorders such as anxiety and depression (1–3). Both anxiety and depression induce changes to the composition and proper functioning of the gut microbiota (4–6). Stress disorders, like anxiety, cause disruptions to the integrity of the gut barrier, which can allow translocation of gut bacterial products, commonly referred to as “leaky gut” (7,8). This likely results in a microbiota-driven proinflammatory response. Animal studies also show an increase of *Bacteroidetes* and a decrease of *Firmicutes* in mice expressing depressive like behavior, indicating a state of microbial dysbiosis (9–11). Interestingly antidepressant medications such as monoamine oxidase inhibitors (MAOIs) and selective serotonin reuptake inhibitors (SSRIs) have antimicrobial properties, suggesting gut modulation as a potential therapeutic target (12,13). Additionally, specific antibiotics show antidepressant properties in both human and rodent studies (14,15).

Animal studies using germ free mice further investigate the role of the microbiome in anxiety and depression. Numerous studies show that germ free mice display lower levels of anxiety-like behavior across multiple models of anxiety: Elevated Plus Maze, Open Field Test, and Light Dark test (16–18). Upon reconstitution of the microbiome in these germ-free mice, anxiety like behavior was only normalized if the reconstitution happened in early life (16,17,19). Reconstitution during adulthood resulted in the persistence of lower anxiety-like behavior. This indicates specific developmental windows in which the microbiome alters neuronal circuitry important for anxiety-like behavior. However, alterations to a healthy microbiome in adulthood can still show alterations to anxiety-like behavior (20). Antibiotic depletion of the microbiome using a broad spectrum antibiotics causes reduced levels of anxiety-like behavior, and once antibiotic administration ceases, the anxiety-like response normalizes as the microbiome replenishes (20). Antibiotic treatment does not affect anxiety-like behavior in germ-free mice, confirming behavioral changes are due to changes in the microbiome. While eliminating harmful bacteria with antibiotics can reduce anxiety-like behavior, it has also been shown that supplementing the microbiome with beneficial bacteria via probiotic treatment can also reduce anxiety-like and depressive-like behavior. Probiotic treatment causes a reduction of anxiety-like and depressive-like behaviors, even in rats experiencing maternal separation during neonatal development, an event known to increase depressive like behavior in adulthood (21,22). The probiotic effects are comparable to results from antidepressant treatment. Clinically, studies show similar antidepressant effects of probiotics with healthy participants displaying less psychological distress when treated with probiotics, and subjects initially scoring highest in depression, showing significant improvement in symptoms (23). Additionally, patients with chronic fatigue syndrome, which has a high comorbidity with anxiety, show a disrupted microbiome composition, and also see improvement of anxiety severity with the treatment of probiotics (24).

Other disruptions to the gut-homeostasis, such as infection and inflammation, are also seen to change anxiety-like behavior. Infections with *C. rodentium* and *C. jejuni* increased anxiety-like behaviors as early as 8 h post infection, lasting up to 2 days (25,26). Interestingly this behavioral change is seen even without a periphery immune response, suggesting that pathogenic bacteria in the gut can produce behavioral changes independently from an immune response (27). Increased anxiety-like behavior is also seen when there is an increase in GI inflammation. This behavioral change was reversed with probiotic treatment.

This relationship between the microbiome and anxiety and depression has great interest in opioid research, as anxiety and depression both have high comorbidity with opioid use disorder, and elevated levels of anxiety and depression are common throughout the stages of opioid use (28). Initial drug is often used as a form of self-medication to alleviate stress and anxieties (29). However, these effects of opioids are short lived, and consumption of drugs can lead to increased depression symptoms instead (30). Once opioid dependence is formed, the abstinence of opioids in the body induces a withdrawal response, which can result in elevated anxiety and depression symptoms, even after the physical symptoms of withdrawal have passed (31–34). For many the severity of anxiety and depression during withdrawal drives continued drug use to alleviate the symptoms (33). The implication of microbiome in these behaviors are especially interesting considering the impact that opioid use has on the gut microbiome.

## Morphine Induced Changes to the Microbiome

Opioid use has been shown to disrupt multiple areas of gut homeostasis. Animal studies show that morphine treatment results in specific changes to the relative abundance of bacteria (35). Morphine exposure, even in the short-term, causes an increased abundance of pathogenic bacteria (*Flavobacterium*, *Enterococcus*, *Fusobacterium*, *Sutterella*, *Clostridium*, *Rikenellaceae*, and *Ruminococcus*). Once tolerance is developed a significant decrease in the abundance of beneficial bacteria (*Lactobacillus* and *Bifidobacterium*) is observed as well (36). This pattern of microbial change is an indication of microbial dysbiosis (37,38). Additional changes in abundance of individual bacteria are seen across morphine exposure time and doses, all indicating the same pattern of increasing of harmful bacteria and decreasing of beneficial bacteria (36,38–46). The functional consequences of the dysbiosis of the microbiome include a decrease in gut motility and an increase in gut-barrier permeability, creating a risk of bacterial translocation and proinflammatory signaling (47).

The diversity of the gut microbiota is also greatly impacted by morphine treatment. Functional and taxonomic diversity of the microbiota are very important for maintaining gut-homeostasis, and a non-diverse microbiota is associated with inflammatory bowel disease and obesity (48,49). Opioid treatment causes a decrease in the alpha diversity of the gut-microbiota, which signifies diminished species richness and a less diverse array of present bacteria within the microbiome (35). Additionally, the beta diversity measuring the similarities or dissimilarities of microbiome composition between groups shows distinct clustering in morphine treated animals as compared to placebo treated controls. This is additional confirmation of a dysbiosis of the gut microbiome caused by morphine treatment.

Studies have been targeting these opioid induced changes in the gut to understand their relevance to the behavioral consequences of opioid use. Tolerance development is the most well studied relationship between the morphine induced dysbiosis and drug related behavior. Germ free mice showed an attenuation of morphine tolerance and the reconstitution of the microbiome *via* a fecal matter transplant (FMT) of a healthy microbiome reinstated the tolerance development, indicating the microbiome is necessary to the development of morphine tolerance (36). Antibiotic depletion of the microbiome in specific pathogen-free (SPF) mice also shows an attenuation of morphine tolerance, however a FMT of a healthy microbiome does not recover the tolerance development. Instead, the FMT of a morphine treated microbiome is needed for the proper development of morphine tolerance. Additionally, treatment with a probiotic cocktail of the bacteria that showed significantly reduced abundance during morphine exposure, both prevents the dysbiosis effects of morphine as well as attenuates morphine tolerance (36). This suggests the state of the microbiome during morphine induced dysbiosis is a requirement for tolerance development. Similar relationships have been seen in other stages of drug use as well. Research shows that antibiotic depletion of the microbiome causes impaired cocaine reward processing, suggesting a need for the microbiome in the rewarding pathways involved in addiction, though no studies have examined the impact of the microbiome in any addiction paradigms for morphine specifically (44). Studies examining addiction paradigms such as Conditioned Place Preference (CPP) show that mice that display higher CPP scores have a unique microbial composition compared to mice that display lower CPP scores (50). A new area of research is the relationship of the morphine induced gut changes on the withdrawal response. However, the limited research shows conflicting results on antibiotics effects on the withdrawal severity. Withdrawal symptoms are seen to both decrease and remain the same depending on the morphine treatment regimen and the specifics of antibiotic treatment (46,51). Though the withdrawal state of morphine use does still show changes to the microbiome as well as neuronal changes that may be linked to gut dysbiosis (52).

The well-studied relationship of the microbiome’s influence on morphine tolerance, as well as emerging evidence of its roles in addition and withdrawal behavior, show that the dysbiosis of the microbiome caused by opioids has an impact on drug related behaviors (36,38,46,51). While researchers are still investigating the extent to which this opioid induced dysbiosis contributes to the severity and development of these behaviors, the importance of this relationship between drug use, microbial changes, and behavior is paramount. Understanding this relationship can lead to the development of gut targeted therapeutic strategies to treat the behavioral symptoms of drug use, an area that is lacking in therapeutic intervention. However, additional research on the mechanisms in which the microbiome is modulating behavior during opioid use is needed. Potential mechanisms are understudied in opioid research, however research with other drugs of misuse has discovered some potential links between microbial and behavioral changes.

## Possible Links Between Microbial and Behavioral Changes

The exact mechanism in which the microbiome influences behavioral changes is unknown, though there are many potential links currently being researched (53–59). One possible connection is the inflammatory response that results from drug induced disruption to the epithelial barrier. The microbial changes that occur during use of opioids, and other drugs of misuse, cause damage to the tight junction proteins (47,60–62). This leads to a compromised integrity of the epithelial lining, allowing for translocation of bacteria. Additionally, there is a higher risk of the translocation of pathogenic bacteria, due to the microbial dysbiosis caused by drug exposure. Host epithelial cells recognize the bacteria and initiate a toll-like receptor (TLR) modulated immune response, resulting in the release of proinflammatory cytokines (63). Studies suggest that these cytokines can cross the blood brain barrier and modulate behavior to contribute to the behavioral consequences of drug use (53,54). However, not all proinflammatory cytokines produce the same behavioral responses. Activation of the TLR4 signaling driven by interleukin 1 beta (IL-1b) results in an increase of CPP and self-administration to cocaine. This indicates that proinflammatory activation drives the addiction process, but conflicting results are found for proinflammatory TNF-a. An increase of TNF-a levels results in a decrease of CPP response to morphine, as well as a decrease of behavioral drug response to both morphine and cocaine. These inconsistent findings suggest the role of inflammation in drug related behavior could depend on the specific cytokine or drug being studied (64–66).

Another potential link is the vagus nerve, as it provides direct communication between the brain and gut. Even though the vagus nerve does not cross the gut epithelial layer and have direct contact with the microbiome, studies have shown that the vagus nerve may be sensitive to signals from the microbiome (55,56). Antimicrobial treatment of the microbiome, resulting in an increase of *Lactobacilli*, modified GABA expression in numerous brain areas and decreased anxiety-like behavior. These findings are thought to be a result of vagal signaling, and additional studies have shown that vagal nerve integrity is crucial to the successful attenuation of anxiety-like behavior by probiotic treatment (21,57). There is also preliminary evidence that shows vagal nerve stimulation facilitates the extinction of drug seeking behavior during the withdrawal process of cocaine treatment (67). While research shows the vagus nerve may be important to the behavioral responses of drug use, there is limited evidence to prove microbiome is relying on the vagus nerve to modulate behavior, or if the microbiome alone can stimulate the vagus nerve enough to produce behavioral changes. Others believe the microbiome modulates behavior *via* hippocampal brain-derived neurotropic factor (BDNF), independent of vagal nerve stimulation (20). Cocaine studies show an epigenetic regulation of BDNF levels during drug use, and BDNF has been shown to mediate cocaine self-administration, and drug seeking (68). Expression of BDNF also changes in response to changes in the microbiome, and these changes are associated with altered behavioral responses to alcohol and cocaine (69–71). A FMT of a microbiome samples of alcohol exposed donors to healthy recipients, resulted in a decrease of BDNF levels in the hippocampus, as well as an increase of anxiety and depressive-like withdrawal behaviors (70). There are many correlations of drug exposure and microbial changes with changes in BDNF expression levels, however causal studies to determine a mechanism in which the microbiome is influencing the BDNF expression have not been done (71).

There is a wealth of data implementing microglial activation as a mechanism that drives microbiome modulated drug related behaviors. It is well documented that the microbiome is crucial for the proper development of microglia (72,73). In fact, germ free mice display deformed microglial cells as well as slight behavioral differences that may be a result of the lack of properly matured microglia (72). On a less severe model, microglial defects can be seen with prolonged antibiotic treatment, and microglial function can be restored with probiotic treatment (72). Also, microglia become significantly more activated during drug exposure (52,58,59,74). Elevated microglial activation occurs as a result of chronic ethanol treatment, and microglia remain overactive throughout long-term ethanol withdrawal (74). Methamphetamine treatment also results in microglia activation, and upon inhibition of microglia cells, locomotor sensitization to methamphetamine attenuated (58). Additionally, high levels of microglial activation in the nucleus accumbens are observed during cocaine treatment (59). This change in microglia activation could be a response to the drug presence itself or a consequence of drug induced microbial changes. The elevation of microglial activation is seen in many brain areas crucial to addiction and reward pathways, and inhibition of microglia has attenuated some behaviors related to drug use. Further research is needed to understand the implications of the gut-microbiome in microglial modulation of drug related behaviors.

## Summary

**FIGURE 1:**
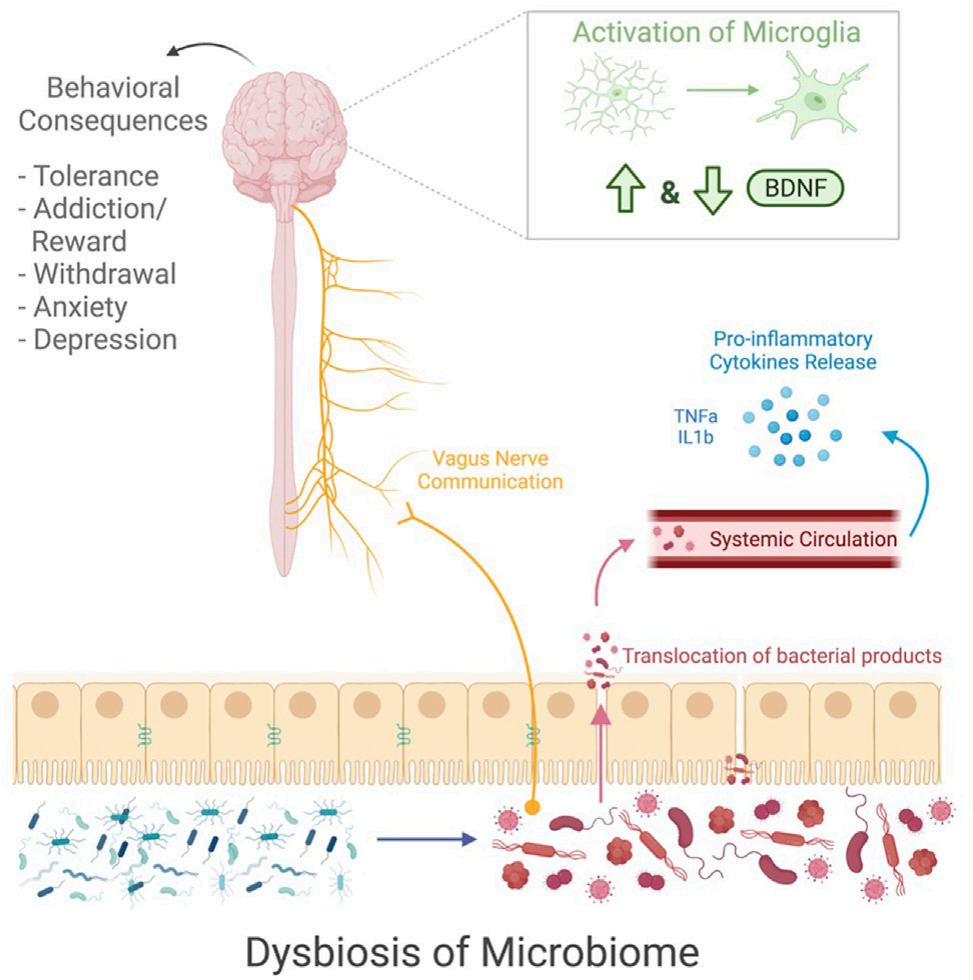
This schematic shows the microbial and neuronal changes that occur during drug use. Exposure to drugs cause a dysbiosis of the microbiome and impaired gut barrier function which leads to the translocation of pathogenic bacteria, resulting in a release of pro-inflammatory cytokines. The vagus nerve sends communications from the gut microbiome to the brain. Additionally, within the brain there is an increased activation of microglia and alterations to BDNF signaling. All of these factors seem to play in a role in drug related behavior, leading to tolerance development, addiction and reward signaling, withdrawal symptoms, anxiety, and depression. Image created with BioRender.com.

Drug use causes dysregulation from the gut to the brain (Figure 1). Research shows a drug induced dysbiosis of the gut microbiome, causing the diverse microbial environment to become overpopulated with pathogenic bacteria (35,36). The consequence of the microbial shift leads to a compromised gut barrier, resulting in a translocation of bacteria that trigger a proinflammatory cytokine release (47). While the microbiome is activating an inflammatory response, it also communicates with the vagus nerve to send signals to the brain (55,56). Additionally, the microbiome may be responsible for the increase in microglial activation as well as dysregulation of BDNF signaling during drug use (52,68,74). All of these factors affected by drug use also have behavioral implications that are relevant to behavioral consequences of drug use. Thus, the dysregulation of these factors during drug use may be during the behavioral consequences of drug use.

In conclusion, there is a lot of evidence showing a relationship between the microbiome and behavior, and the microbiome undergoes substantial changes when exposed to opioids that may further modulate drug related behaviors, such as reward processing, tolerance, withdrawal, anxiety, and depression. However, the exact mechanism between microbial changes and behavior is still not understood, especially for opioids specifically. The collective literature for drugs of misuse, provide potential links between the microbiome and the brain: inflammation, the vagus nerve, BDNF, and microglia. These are areas that need additional research for opioids, as well as every drug individually, to determine how the gut and brain are communicating and regulating drug related behaviors. Understanding this relationship could lead to potential treatment options for the psychiatric symptoms of SUD and provide an easier path out of the cycle of addition.
